# Supplementary material for: The Robust Self-Assembling Tubular Nanostructures Formed by gp053 from Phage vB_EcoM_FV3
Source: Viruses. 2019 Jan 11;11(1):50. doi: 10.3390/v11010050 (PMC6357053; doi:10.3390/v11010050)
Supplement: Supplementary file 1 [file viruses-11-00050-s001.pdf]

## SUPPLEMENTARY MATERIAL

**Table S1.** Oligonucleotides used in this work. The restriction sites are underlined.

| Primer name | Primer sequence | Restriction enzyme |
|---|---|---|
| FV53_NdeI_F1 | GGAAACATATGCCATATTTAGATAAAGTG | NdeI |
| FV53_BamHI_R1 | TTGAGGATCCATCTAGTCTTCCTTATTC | BamHI |
| FV53_XhoI_R1 | CATCTAGTCTTCCTCGAGTTCAGTCATTG | XhoI |
| FV53_Nde10F | TAAAGTGGTACATATGACAGTTAAC | NdeI |
| FV53_Bam10R | CAGCATTGGTGGATCCTTAACCAAC | BamHI |
| FV53_Xho10R | CAGCATTGGTGGACTCGAGACCAAC | XhoI |
| FV53_Nde20F | CCCAGCCTATTCATATGGTAGGTTTC | NdeI |
| FV53_Bam20RN | GTCCAGAAGGATCCTTTACCGGATACG | BamHI |
| FV53_Xho20R | GAAGGACGTTGATCTCGAGACGAAC | XhoI |
| FV53_Nde25F | CTGTAGGTTTCCATATGCCGCTG) | NdeI |
| FV53_Bam25R | GTTGATCCGGATCCGAACTTAGTGC | BamHI |
| FV53_Xho25R | GATCCGGATACGCTCGAGGTGCAG | XhoI |
| FV53_Nde30F | GAAACTCCGCTGCATATGGCTATCC | NdeI |
| FV53_Bam30R | CGAAGTGCGGATCCTTGTTTTACAC | BamHI |
| FV53_Xho30R | GAAGTGCAGGGCGTCTCGAGACACCAG | XhoI |
| FV3_Bam50R | CTGATTGGATCCGATCTATGCACG | BamHI |
| FV3_Bam75R | CTGTCAGGATCCCTCAATTCTTAATG | BamHI |
| FV3_Bam100R | CGGAAGATCAGGATCCGACTACTTCATG | BamHI |
| FV3_Bam150R | CAACACCGTGGATCCTTCAGTAGAAG | BamHI |
| FV3_Bam200R | CACCTATGATAGGATCCTACGGAAAATC | BamHI |
| FV3_m_N_C_F | GATATACATATGGCTAGCACAGGTACCCCA TATTTAGATAAAGTGG | NdeI, NheI, Acc65I |
| FV3_m_N_C_R | GTGGTGCTCGAGTTACGTACGTGTACTAGTT TCAGTCATTGCAACAGC | XhoI, Pfl23II, BcuI |

**Table S2.** Constructs of gp053.

| Mutant | Primers used for PCR fragment amplification | Expression vector | Amino acid residues, MW (kDa) | Description of self-assembling structures |
|---|---|---|---|---|
| Full-length gp053 | FV53_NdeI_F1<br>FV53_BamHI_R1 | pET-21a | 458 (50.2) | Regular polysheaths, length up to ~1000 nm, width 27,8±2,7 nm. |
| gp053_N-his | FV53_NdeI_F1<br>FV53_BamHI_R1 | pET-16b | 479 (52.8) | Regular polysheaths, length up to ~300 nm, width ~28 nm. |
| gp053_C-his | FV53_NdeI_F1<br>FV53_XhoI_R1 | pET-21a | 466 (51.3) | Regular polysheaths, length up to ~1000 nm, width ~28 nm. |
| gp053_NΔ9 | FV53_Nde10F<br>FV53_BamHI_R1 | pET-21a | 449 (49.2) | Regular polysheaths, length up to ~600 nm, width ~28 nm. |
| gp053_CΔ11 | FV53_NdeI_F1<br>FV53_Bam10R | pET-21a | 447 (49.1) | Regular polysheaths, length up to ~600 nm, width ~28 nm. |
| gp053_NΔ9_CΔ11 | FV53_Nde10F<br>FV53_Bam10R | pET-21a | 438 (48.1) | Regular polysheaths, length up to ~500 nm, width ~28 nm. |
| gp053_NΔ9-his_CΔ11 | FV53_Nde10F<br>FV53_Bam10R | pET-16b | 459 (50.6) | Regular polysheaths, length up to ~800 nm, width ~28 nm. |
| gp053_NΔ9_CΔ11-his | FV53_Nde10F<br>FV53_Xho10R | pET-21a | 446 (49.2) | Regular polysheaths, length up to ~800 nm, width ~28 nm. |
| gp053_NΔ20 | FV53_Nde20F<br>FV53_BamHI_R1 | pET-21a | 438 (48.1) | Regular polysheaths, length up to ~500 nm, width ~28 nm. |
| gp053_NΔ20_CΔ20 | FV53_Nde20F<br>FV53_Bam20RN | pET-21a | 418 (46.0) | Regular polysheaths, length up to ~600 nm, width ~28 nm. |
| gp053_NΔ20-his_CΔ20 | FV53_Nde20F<br>FV53_Bam20RN | pET-16b | 439 (48.5) | Regular polysheaths, length up to ~1000 nm, width ~28 nm. |
| gp053_NΔ20_CΔ20-his | FV53_Nde20F<br>FV53_Xho20R | pET-21a | 424 (46.8) | Regular polysheaths, length up to ~1000 nm, width ~28 nm. |
| gp053_NΔ25_CΔ25 | FV53_Nde25F<br>FV53_Bam25R | pET-21a | 408 (44.8) | Regular polysheaths, length up to ~800 nm, width ~28 nm. |
| gp053_NΔ25-his_CΔ25 | FV53_Nde25F | pET-16b | 429 (47.3) | Regular polysheaths, length up to ~800 |

| | | | | |
|---|---|---|---|---|
| | FV53_Bam25RN | | | nm), width ~28 nm. |
| gp053_NΔ25_CΔ25-his | FV53_Nde25F<br>FV53_Xho25R | pET-21a | 416 (45.9) | Regular polysheaths, length up to ~800 nm, width ~28 nm. |
| gp053_NΔ29 | FV53_Nde30F<br>FV53_BamHI_R1 | pET-21a | 429 (47.1) | Soluble unordered protein ribbons |
| gp053_NΔ29_CΔ31 | FV53_Nde30F<br>FV53_Bam30R | pET-21a | 398 (43.6) | Soluble unordered protein ribbons |
| gp053_NΔ29-his_CΔ31 | FV53_Nde30F<br>FV53_Bam30R | pET-16b | 419 (46.1) | Insoluble protein |
| gp053_CΔ31 | FV53_NdeI_F1<br>FV53_Bam30R | pET-21a | 427 (46.8) | Regular polysheaths, length up to ~600 nm, width ~28 nm. |
| gp053_CΔ51 | FV53_NdeI_F1<br>FV53_Bam50R | pET-21a | 407 (44.6) | Regular polysheaths, length up to ~600 nm, width ~28 nm. |
| gp053_CΔ76 | FV53_NdeI_F1<br>FV53_Bam75R | pET-21a | 382 (41.9) | Regular polysheaths, length up to ~600 nm, width ~28 nm. |
| gp053_CΔ100 | FV53_NdeI_F1<br>FV53_Bam100R | pET-21a | 358 (39.3) | Regular polysheaths, length up to ~3000 nm, width ~32 nm. |
| gp053_CΔ152 | FV53_NdeI_F1<br>FV53_Bam150R | pET-21a | 306 (33.2) | Soluble unordered protein ribbons |
| gp053_CΔ200 | FV53_NdeI_F1<br>FV53_Bam200R | pET-21a | 258 (28.0) | Soluble unordered protein ribbons |
| gp053_C-strep | FV53_NdeI_F1<br>Pfl23Strep | pET21a | 468 (51.54) | Regular polysheaths, length up to ~500 nm, width ~28 nm. |
| gp053_NΔ25_C-strep | FV53_Nde25F<br>Pfl23Strep | pET21a | 443 (48.84) | Insoluble protein |
| gp053_N_C_mon | FV3_m_N_C_F<br>FV3_m_N_C_R | pET21a | 468 (51.21) | Regular polysheaths, length up to ~500 nm, width ~28 nm. |
| gp053_N_C_dim | FV3_m_N_C_F<br>FV3_m_N_C_R | pET21a | 930 (101.75) | Irregular polysheaths, length up to ~200 nm after 48 hours of incubation *in vitro* at 22°C. |
| gp053_N_C_trim | FV3_m_N_C_F<br>FV3_m_N_C_R | pET21a | 1392 (152.29) | Irregular polysheaths, length up to ~200 nm after 48 hours of incubation *in vitro* at 22°C. |

| | | | | |
|---|---|---|---|---|
| gp053_N_C_tetr | FV3_m_N_C_F<br>FV3_m_N_C_R | pET21a | 1854 (202.83) | Irregular polysheaths, length up to ~150 nm after 48 hours of incubation *in vitro* at 22°C. |
| gp053_N_C_pent | FV3_m_N_C_F<br>FV3_m_N_C_R | pET21a | 2316 (253.38) | Irregular polysheaths, length up to ~200 nm after 48 hours of incubation *in vitro* at 22°C. |
| gp053_N_C_hex | FV3_m_N_C_F<br>FV3_m_N_C_R | pET21a | 2778 (303.92) | Irregular polysheaths, length up to ~150 nm after 48 hours of incubation *in vitro* at 22°C. |

**Supplementary Table S3.** Homology of gp053 from FV3 with proteins from other bacteriophages.

| Significant match<br>protein accession number, organism (taxonomic lineage) | Identity aa %/ similarity aa% (length of the overlapping segment) | E value |
|---|---|---|
| AKO61920.1, *Escherichia* phage APCEc02 (*Vequintavirinae*, V5virus) | 99/99 (458) | 0.0 |
| ATW61401.1, phage 203 (*Vequintavirinae*, V5virus) | 99/99 (458) | 0.0 |
| YP_002003555.1, *Escherichia* phage V5 (*Vequintavirinae*, V5virus) | 99/99 (458) | 0.0 |
| YP_009177499.1, *Escherichia* phage vB_EcoM_FFH2 (*Vequintavirinae*, V5virus) | 99/99 (458) | 0.0 |
| YP_008530297.1, *Escherichia* phage JES2013 (*Vequintavirinae*, V5virus) | 99/99 (458) | 0.0 |
| ASJ80411.1, *Escherichia* phage V18 (*Vequintavirinae*, V5virus) | 98/99 (458) | 0.0 |
| AKU44144.1, *Escherichia* phage Murica (*Vequintavirinae*, V5virus) | 98/99 (458) | 0.0 |
| AKE47028.1, *Escherichia coli* O157 typing phage 14 (*Vequintavirinae*, V5virus) | 98/98 (458) | 0.0 |
| AVJ51863.1, *Escherichia* phage PDX (*Vequintavirinae*, V5virus) | 96/98 (458) | 0.0 |
| AXU22620.1, *Escherichia* phage vB_EcoM-Pr121LW (*Vequintavirinae*, V5virus) | 96/98 (458) | 0.0 |
| AXC42731.1, *Escherichia* phage LL12 (*Vequintavirinae*, V5virus) | 96/98 (458) | 0.0 |
| YP_006987047.1, *Cronobacter* phage vB_CsaM_GAP31 (*Vequintavirinae*, Se1virus) | 60/71 (468) | 0.0 |

| | | |
|---|---|---|
| YP_009111160.1, *Escherichia* phage Av-05 (unclassified myovirus) | 59/72 (468) | 0.0 |
| YP_004893867.1, *Salmonella* phage PVP-SE1 (*Vequintavirinae*, Se1virus) | 59/73 (469) | 0.0 |
| YP_008857275.1, Enterobacteria phage 4MG (*Vequintavirinae*, Se1virus) | 59/71 (469) | 0.0 |
| ARB12543.1, *Klebsiella* phage vB_KpnP_BIS47 (unclassified bacterial virus) | 59/71 (469) | 0.0 |
| YP_009187676.1, *Klebsiella* phage vB_KpnM_KB57 (unclassified myovirus) | 57/68 (493) | 0.0 |
| AXN57735.1, *Acinetobacter* phage ABPH49 (unclassified myovirus) | 57/69 (468) | 8e-174 |
| ARB11491.1, *Pectobacterium* phage vB_PatM_CB7 (*Vequintavirinae*, Cr3virus) | 53/66 (464) | 4e-159 |
| YP_006383031.1, *Cronobacter* phage CR3 (*Vequintavirinae*, Cr3virus) | 53/66 (464) | 7e-158 |
| YP_009042253.1, *Cronobacter* phage CR8 (*Vequintavirinae*, Cr3virus) | 53/66 (464) | 1e-157 |
| YP_009014977.1, *Cronobacter* phage CR9 (*Vequintavirinae*, Cr3virus) | 53/66 (462) | 1e-157 |
| ATS93415.1, *Pectobacterium* phage DU_PP_I (*Vequintavirinae*, Cr3virus) | 53/65 (464) | 2e-157 |
| YP_007392684.1, *Pectobacterium* phage phiTE (*Vequintavirinae*, Cr3virus) | 52/66 (462) | 3e-155 |

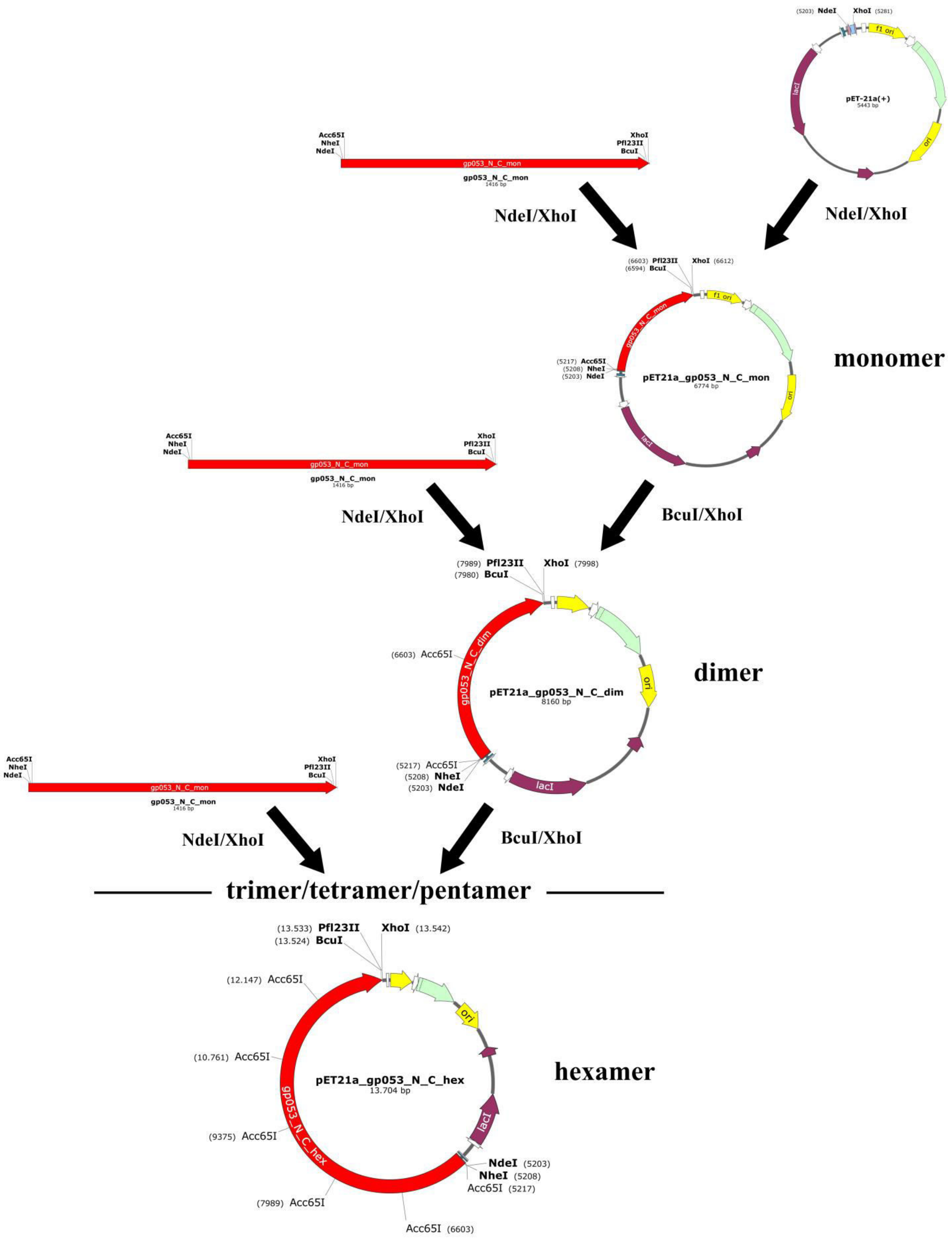

(5203) NdeI
XhoI (5281)
f1 ori
lacI
ori
pET-21a(+)
5443 bp
Acc65I
NheI
NdeI
XhoI
Pfl23II
BcuI
gp053_N_C_mon
gp053_N_C_mon
1416 bp
NdeI/XhoI
NdeI/XhoI
(6603) Pfl23II
(6594) BcuI
XhoI (6612)
(5217) Acc65I
(5208) NheI
(5203) NdeI
pET21a_gp053_N_C_mon
6774 bp
monomer
BcuI/XhoI
(7989) Pfl23II
(7980) BcuI
XhoI (7998)
(6603) Acc65I
gp053_N_C_dim
pET21a_gp053_N_C_dim
8160 bp
(5217) Acc65I
(5208) NheI
(5203) NdeI
dimer
trimer/tetramer/pentamer
(13.533) Pfl23II
(13.524) BcuI
XhoI (13.542)
(12.147) Acc65I
(10.761) Acc65I
(9375) Acc65I
(7989) Acc65I
Acc65I (6603)
gp053_N_C_hex
pET21a_gp053_N_C_hex
13.704 bp
NdeI (5203)
NheI (5208)
Acc65I (5217)
hexamer

**Supplementary Figure S1.** Schematic representation of construction of gp053 oligomers. The PCR fragment of gene *053* mutant gp053_N_C_mon was obtained by amplification of phage DNA using FV3_m_N_C_F and FV3_m_N_C_R primers presented in Supplementary Table S1. Restriction endonucleases, which were used to obtain restricted fragments and cloning vectors, are shown beside the arrows.

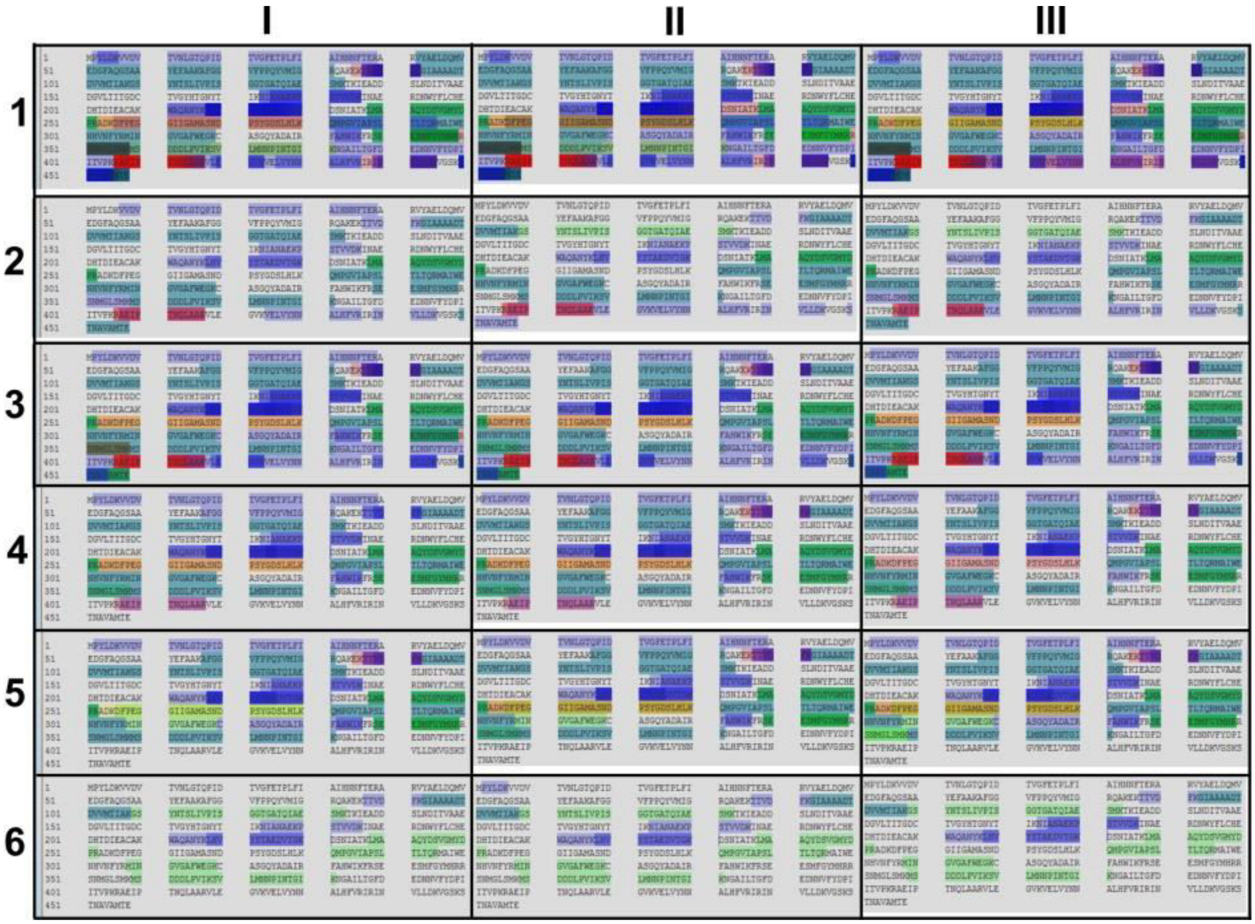


**Supplementary Figure S2.** The protein sequence coverage map obtained from the PLGS-processed $MS^E$ data. Rows (1-6) indicate the number of protein band, excised from the gel, columns (I-III) indicate three technical replicates of LC-MS/MS. Regions of the protein sequence that match peptides are highlighted in color: blue– matches to a peptide, red– matches to a partial peptide, green– matches to a modified peptide, yellow– matches to a partial modified peptide.
